# Suicidal Behavior in Emergency Child and Adolescent Psychiatric Service Users Before and During the 16 Months of the COVID-19 Pandemic

**DOI:** 10.3389/fpsyt.2022.893040

**Published:** 2022-05-11

**Authors:** Barbara Kirič, Lara Leben Novak, Petra Lušicky, Maja Drobnič Radobuljac

**Affiliations:** ^1^Center for Mental Health, University Psychiatric Clinic Ljubljana, Ljubljana, Slovenia; ^2^Faculty of Medicine, University of Ljubljana, Ljubljana, Slovenia; ^3^Community Health Center Kranj, Kranj, Slovenia

**Keywords:** COVID-19, pandemic (COVID-19), suicidal thoughts and behavior (STB), attempted suicide, emergency psychiatric service, child and adolescent

## Abstract

**Background:**

Slovenia is among the countries with the highest suicide rates in the world. The COVID-19 pandemic has had a significant impact on the mental health of children and adolescents. Our hypothesis is that the school closure during the pandemic with a gradual transfer to virtual schooling had an important impact on children's and adolescents' suicidal behavior. Therefore, we aimed to determine possible changes in the frequency of assessments as well as frequency and severity of suicidal behavior in the population of Slovene children and adolescents seeking emergency psychiatric help in correlation with the progression of the pandemic and online schooling.

**Methods:**

We performed a retrospective observational analysis of medical records of all children and adolescents referred to the only 24-h emergency in- and outpatient child and adolescent psychiatry service in Slovenia from March 2019 through the end of July 2021. We extracted number of assessments, number of patients with suicidal ideation and with attempted suicide. A comparison between the same periods prior to the pandemic and during the pandemic was made. The months of school closure due to the COVID-19 restriction measures and the months without closures were also compared.

**Results:**

During this period, 1966 children and adolescents were assessed. There was no statistically significant difference in the observed frequency of emergency visits when we compared all the months with to all the months without school closures, or when individual corresponding months with and without school closures were compared. However, there were statistically significantly more patients with suicidal ideation [*t*(16) = −2.739, *p* = 0.015; W = 25.0, *p* = 0.016] and patients who had attempted suicide [*t*(16)= −3.412, *p*= 0.004; W = 14.5, *p* =0.006] during the pandemic as individually compared to the corresponding pre-pandemic months.

**Conclusions:**

Our results show that the number of Slovene children and adolescents who required emergency psychiatric help with suicidality and attempted suicide increased during the COVID-19 pandemic. The increase was shown only after the first year of the pandemic. The observed increase did not appear to directly correspond to the school closures, but was more likely related to the duration of the pandemic.

## Introduction

The new coronavirus outbreak, declared as an epidemic by the World Health Organization on March 11, 2020, considerably changed the lives of the population (1). Many countries soon undertook measures to limit the spread of the disease, including recommendations for staying at home, closing schools, and social distancing, which significantly affected the mental health of children and adolescents (2).

A review, published in August 2020, examined the impact of the pandemic on the mental health of children and adolescents. Increased fear of infection and clinginess were found in younger children. Changes in routines, uncertainty and anxiety related to disruption in their education were seen in adolescents, as well as hoarding behavior, increased use of the internet, and limited ability to report violence and abuse. Children with special needs, neurodevelopmental difficulties or underprivileged youth were more vulnerable (3). On the other hand, a study by Penner et al. showed significant reduction in internalizing, attention, externalizing, and total mental health problems 1 month after school closures and start of virtual schooling in a predominantly racial and ethnic minority groups sample, who had had elevated levels of mental health problems before the pandemic. For some groups, this points to protective aspects of stay-at-home measures. Increased family time, decreased peer and academic stressors, community factors, more flexible routines at home, and the timeline of the conducted research were identified as possible protective factors (4). In October and November 2020, the Centers for Disease Control and Prevention (CDC) in the United States examined the differences in child and parent indicators of well-being according to the child's mode of schooling. Negative indicators were reported to a greater extent by parents of children receiving virtual or combined instruction than parents whose children received in-person instruction. Parents of the first group more frequently reported that their child's mental or emotional health worsened and that they spent less time outside, in-person with friends, or being physically active (5). A survey published in September 2021 found worse mental health outcomes in older children and children of low-income families that attended school remotely than those who attended school in person. Younger children who attended school remotely had comparable or slightly better mental health outcomes than those who attended school in person (6).

The COVID-19 pandemic affected child and adolescent psychiatry services as well. There was a decrease in the number of outpatient visits and hospital admissions reported in the longitudinal survey by European Society for Child and Adolescent Psychiatry (ESCAP) in 22 European countries at the beginning of the pandemic in March and April 2020. At that time, the overall perception of COVID-19 crisis was perceived as ≫medium≪. Almost a year later, in February and March 2021, the perceived impact increased to ≫strong≪ or ≫extreme≪, and there was also a substantial increase in referrals or requests for assessments. The impact on psychopathology appeared substantially more marked during the second phase of the study, particularly for anxiety disorders, major depressive episodes, eating disorders and suicidal crises (7). Similarly, a study by Fitzpatrick et al. reported high levels of perceived need for mental health services by children/adolescents and their caregivers. Raised levels of mental health symptoms in caregivers and children/adolescents with both internalizing and externalizing symptoms were observed (2).

So far, the reported data on suicidal behavior is showing mixed results. In Japan, suicide rates among children and adolescents (up to 20 years old) were not significantly affected during the first wave of the pandemic (8). In a French study, Mourouvaye et al. found a 50% decrease in the incidence of suicidal behavior in children and adolescents during the COVID-19 lockdown between March and May 2020. This was attributed to reduced help-seeking and a global decrease in hospital admission rates, as well as environmental and cognitive factors (9). On the other hand, Hill et al. found higher positive screen results for recent suicide attempts in a pediatric emergency department (ED) in February, March, April and July 2020, compared to the same months in the previous year. The results were not uniformly higher after the outbreak, which could be related to the time course of the pandemic and the generally reduced rate of ED visits, with elevated numbers of cases of suicidal ideation or attempts due to the increased overall severity of cases (10). A study of hospitalized adolescents, aged 11–18 years, found elevated rates of suicidal ideation and suicidal attempts during the pandemic as well, compared to the data from the same months in a year before (11). A large recent retrospective Swiss chart review study, reporting on emergency child and adolescent psychiatry assessments and hospitalizations, reported an initial drop of in-person assessments and hospitalizations during the first wave of the epidemic, only to observe a progressive rise from the end of the 2020 summer holidays until the end of June 2021 (the end of the study). Similar was observed for suicidal ideation and self-harm behaviors. They reported a slight drop in the number of outpatient assessments of patients with suicidal ideation and self-harm in March and April 2020 as compared to 2019, however they observed a twofold rise in the same 2 months in 2021 (12).

Slovenia is a country with traditionally high suicide rate. A 1999 study including 4706 Slovene high-school students reported, that by the age of 19 years 10% had had attempted suicide and 44% had experienced suicide ideation. When compared to the Dutch population, Slovene prevalences were more than three times higher for attempted suicide and twice as high for suicidal ideation (13). Similar prevalences of attempted suicide and suicidal ideation as in 1999 were subsequently reported on a smaller sample of Slovene high-school students in 2009 (14). However, these behaviors were only studied in the general population. Up to now, no study reported the prevalence of suicidal behaviors (ideation or attempts) in Slovene in- or outpatient emergency child and adolescent psychiatric service users.

Slovenia declared the COVID-19 epidemic on March 12, 2020 (15). The mode of school instruction in the remainder of the school year and during the subsequent school year varied from in-person to virtual schooling (as well as alternating both modes), depending on the epidemic progression, the restrictive measures issued, and the students' age group (16, 17). From the end of 2020 until the end of 2021 the Slovenian National Institute for Public Health conducted several surveys on adults that reported a decrease in perceived mental health, which was disproportionally severe for the younger population (aged 18–29 years) (18).

There was no research however, on the impact of the COVID-19 pandemic on the most endangering mental health symptoms in Slovene children and adolescents so far. The present study aimed to determine possible changes in the frequency of assessments and admissions and severity of suicidal behaviors in the population of Slovene children and adolescents seeking emergency psychiatric services.

## Methods

### Sample

We performed a retrospective observational analysis of medical records of all the children and adolescents referred to the only 24-h emergency in- and outpatient child and adolescent psychiatry service in Slovenia from March 2019 to the end of July 2021.

### Setting

Emergency child and adolescent psychiatric help in Slovenia is organized in three distinct centers in two separate geographic parts of the country. The first two centers offer assessments during working h (Monday-Friday, 8–15 h). The third, located in the University Psychiatric Clinic Ljubljana, offers emergency outpatient assessments in a clinic attached to the only Slovene Intensive Child and Adolescent Psychiatry Unit. As such it offers assessment and treatment by a child and adolescent psychiatrist 24/7 for the entire country and provides more than 50% of emergency outpatient and inpatient treatments (19). Children and adolescents aged 0–19 years are referred for assessment by their pediatrician or other emergency doctor, however, they can be examined by self or parents' referral as well.

All the data was extracted from the written and electronic medical records. The number of emergency outpatient assessments and admissions was recorded separately for each month from March 2019 until September 2021. For all the patients assessed or admitted from March 2019 until the end of July 2021 the medical history reports and mental state examinations were examined for age, gender, suicidal ideation at assessment, past and recent attempted suicide (as a reason for referral). Suicidal ideation, and current and past attempted suicides are routinely screened for in every psychiatric examination and recorded in the patients' medical history. Suicidal ideation was recorded as present when the patient reported he/she wished they were dead, thought or planned about killing themselves. A current attempted suicide was recorded when the patient was assessed after a recent self-inflicted action made with an intent of dying (regardless of the chosen method) or after something or somebody interrupted such an event. A past attempted suicide was recorded if a patient reported one or more attempts or interrupted attempts in the past.

The study was approved by the Ethical Review Board of the University Psychiatric Clinic Ljubljana in 2021.

### The Schedule of Regular and Pandemic School Closures

A school closure with a gradual transfer to virtual schooling for children and adolescents began on March 16th and lasted until May 18th, 2020, when children aged 6–9 years and high school seniors (18 years old) returned to in-person schooling, or until May 25th when all other primary and secondary school children returned to school (16). In the subsequent school year, the schools were closed and provided online instructions from October 26th, 2020 until January 25th, 2021 for 6–9-year-olds and until February 15th, 2021 for the rest of the children and adolescents. Adolescents attending high school were schooled online from October 10th–February 15th, 2021, when seniors returned to in-person schooling, while others took bi-weekly turns (alternating between in-person and online schooling) until May 17th, 2021. For a brief period of time, between April 1–11th, 2021, all children and adolescents returned to online schooling (17).

The regular school holidays in Slovenia are as follows: October 26th–November 1st, December 25th–January 2nd, February (1 week, varies each year), April 27th–May 2nd and June 25th–September 1st (20).

### Statistical Analysis

Analyses were performed in the statistical package IBM® SPSS® Statistics Version 28.0.0.0 (© IBM Corporation and its licensors 1989, 2021), and α = 0.05 level of significance was used.

The descriptive comparisons between genders were made using independent samples *t*-test (continuous variables) and Pearson chi-square or Fisher's exact test predictor (categorical variables).

Two independent samples comparisons were conducted between the months where the schools were closed due to the COVID-19 restriction measures for at least 1 day and the months without closures.

The parametric independent samples *t*-test and the non-parametric Mann-Whitney U test were used. The comparisons were made for the total number of all emergency patients and separately for admitted patients, outpatients, patients who were suicidal at the assessment, and those who were assessed after attempting suicide.

The same months were also compared in pairs with the paired samples *t*-test and Wilcoxon signed-rank test: months during COVID-19 pandemic (from March 2020) vs. the same months before the COVID-19 epidemic (through February 2020). As the available data was from March 2019 up to July 2021 (for number of emergency admissions, outpatient assessments and combined emergencies, including September 2021), only exactly 1 year of COVID-19-free months was available. This means that both March 2020 and March 2021 were individually compared to the only March before COVID-19 (March 2019). School closure played no role in this last analysis.

## Results

During this period, 1966 children and adolescents were assessed; more than 99.9 % of the participants were white European. All the assessed suicidal behavior was statistically significantly more frequent in females than males (Table 1).

**Table 1:** Descriptive statistics of all the consecutive patients assessed as outpatients and admitted to the hospital from March 1^st^ 2019 until July 31^st^ 2021.

	**Female**	**Male**	**All**	***p***
Number of patients	1362 (69.3)	604 (30.7)	1966	
Age (years ± SD)	16.30 ± 1.53	16.21 ± 2.10	16.28 ± 1.72 (min 6.9, max 25.2^*^)	0.391
Emergency outpatient	894 (68.7)	407 (31.3)	1301	0.450
Emergency inpatient	468 (70.4)	197 (29.6)	665	
Attempted suicide before assessment^a^	178 (13.1)	49 (8.1)	227 (11.7)	**0.003**
Suicidal at assessment^b^	834 (61.2)	237 (39.2)	1071 (54.5)	**<0.001**
Ever attempted suicide^c^	471 (35.2)	99 (16.6)	570 (29.5)	**<0.001**
Never attempted suicide^c^	866 (64.8)	498 (83.4)	1364 (70.5)	**<0.001**
Attempted suicide only in the past^a^	368 (27.0)	64 (10.6)	432 (22.0)	**<0.001**

*Data are presented as n (%) unless stated otherwise. The statistics used were χ^2^ and Student's t-test; bold means the differences are statistically significant. Number of missing data*,

a *31 cases*;

b *29 cases*;

c *32 cases*.

* *The emergency out- and inpatient services provide for the patients up to the age of 19 years. In only one case (aged 25.2 years) an exception was made upon a decision of an extended medical board to arrange a specific type of emergency treatment in a secure child and adolescent psychiatric hospital department*.

**Figure 1:**
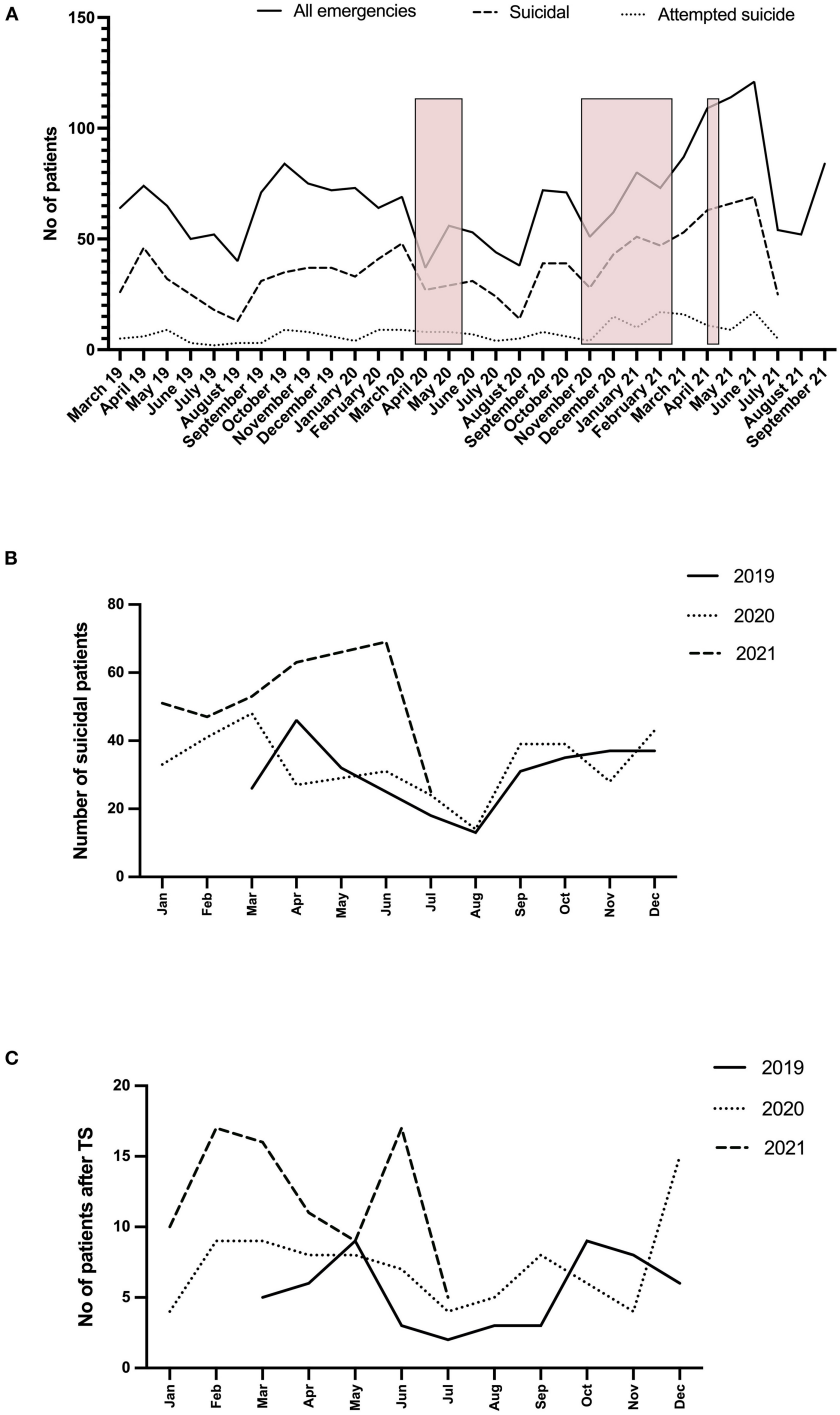
Time course of monthly emergency service assessments (outpatient and inpatient) from the beginning of March 2019 until the end of July 2021. **(A)** The number of all the assessed patients (through the end of September 2021), suicidal patients (suicidal ideation + attempted suicide) and patients after attempted suicides for given months. The pink squares represent the months with school closures due to the pandemic restriction measures. **(B)** Comparison of the number of suicidal patients (suicidal ideation + attempted suicide) for the comparative months over three consecutive years – before and during the COVID-19 pandemic. **(C)** Comparison of the number of patients assessed after attempting suicide (TS) for the comparative months over three consecutive years – before and during the COVID-19 pandemic.

There were no statistically significant differences in any of the observed frequencies when months with and without school closures were compared, nor when comparing individual corresponding months with and without school closures (Figure 1).

However, there were statistically significantly more patients with suicidal ideation [*t*(16)= −2.739, *p* = 0.015; W = 25.0, *p* = 0.016] and attempted suicide [*t*(16) = −3.412, *p* = 0.004; W = 14.5, *p* = 0.006] during the pandemic as individually compared to the corresponding pre-pandemic months (Figure 1, Tables 2, 3).

**Table 2:** Comparison of the number of suicidal patients (with suicidal ideation or after attempting suicide) on individual corresponding months during the years 2019, 2020, and 2021.

	**Jan**	**Feb**	**Mar**	**Apr**	**May**	**June**	**July**	**Aug**	**Sept**	**Oct**	**Nov**	**Dec**
**2019**			26	46	32	25	18	13	31	35	37	37
**2020**	33	41	48	27	29	31	24	14	39	39	28	43
**2021**	51	47	53	63	66	69	26					

*The available data are from March 2019 until July 2021. Missing data for 29 cases out of 1966 of all the assessed patients in the observed period (suicidal and non-suicidal)*.

**Table 3:** Comparison of the number of patients after attempted suicides on individual corresponding months during the years 2019, 2020 and 2021.

	**Jan**	**Feb**	**Mar**	**Apr**	**Maj**	**Jun**	**Jul**	**Aug**	**Sept**	**Oct**	**Nov**	**Dec**
**2019**			5	6	9	3	2	3	3	9	8	6
**2020**	4	9	9	8	8	7	4	5	8	6	4	15
**2021**	10	17	16	11	9	17	6					

*The available data are from March 2019 until July 2021. Missing data for 31 cases out of 1966 of all the assessed patients in the observed period (suicidal and non-suicidal)*.

## Discussion

This is a retrospective chart review study aiming to determine possible changes in the frequency of emergency assessments, and frequency and severity of suicidal behavior in the population of Slovene children and adolescents seeking emergency psychiatric services during the COVID-19 pandemic.

Even though we weren't able to show (however perceived) an increase in all emergencies, our results show that the number of children and adolescents who required emergency psychiatric help for suicidality and attempted suicide in the only 24-h emergency child and adolescent psychiatric service in Slovenia significantly increased during the COVID-19 pandemic compared to the pre-pandemic months. After a small decline, the increase was shown only after the first year of the pandemic, which corresponds with the results of previous studies (21–23). Similar to other reports, the trend of increasing frequency of emergency visits due to suicidality persisted as the pandemic progressed (21). An initial decline during the first wave of the pandemic is a result similar to the reports from Japan, France and Switzerland (8, 9, 12, 24). To point out a recent study from Zurich, similar to our results, noted a brief decline in the emergency consultations in the initial 2 months of the first lockdown and a stable increase of consultations and frequency of patients with suicidality and self-harm from August 2020 onward (12). Thus, the increase in suicidality did not seem to directly correspond to the school closures but more likely to the duration of the pandemic. It is important to note, that the overall number of visits to their emergency child and adolescent psychiatry service increased as well, even though all the other child and adolescent mental health services were operating at least on-line after the first wave (7, 12).

Similar to other studies on adolescent suicidal behavior and psychiatric service use (12–14), our results confirmed that the observed behaviors were more frequent in females.

As the COVID-19 pandemic continued, potential social, economic and health stressors increased, thus contributing to psychological distress, and together increasing the risk factors for self-harm. Young females are a group that can be particularly affected due to social isolation, leading to a higher level of loneliness, anxiety and stress. Adolescents generally have a weaker ability to cope with stressful situations than adults and are prone to react impulsively and emotionally. As a result, pandemic-related distress can lead to increased suicidal behavior and a generally increased number of referrals for assessments in this age group (12). During the pandemic lockdowns, many adolescents spent even more time on social media platforms. Even though one is not allowed to share content depicting, promoting, normalizing or encouraging others to partake in dangerous activities that may lead to serious injury or death, these types of materials (e.g., comments, pictures, videos) are still posted, providing fodder for adolescent imitative behavior and influencing suicidal behavior (25).

During the first wave of the lockdowns, academic pressures might have been reduced due to virtual schooling and general expectations for a close end to the pandemic. Students were able to spend more time on schoolwork while simultaneously perceiving lower academic demands, including decreased numbers of officially required school assessments. Most parents were at home at least partially due to the general public lockdown and able to provide help, support, encouragement and structure. In the subsequent pandemic waves, the hybrid model of schooling was established and the perceived school demands increased along with increased demands on their parents due to work and/or potential economic instability. Stress related to studies, grades, and difficulties in attending online classes due to inadequate technical or adult support is likely one of the important risk factors contributing to increased adolescent suicidal behavior during the COVID-19 pandemic (23). However, the present study was not able to test those assumptions.

Our study had several limitations. The data was collected in only one emergency child and adolescent psychiatry unit in the country. While the service itself covers the majority of the country's emergency assessments and admissions, the remaining two units were not included in the study. Therefore, caution is needed in generalizing from the presented data to other emergency child and adolescent psychiatry services or to the general population. As this was a retrospective observational analysis, we weren't able to assess and control for multiple factors influencing suicidal behavior in adolescents. This, however, was not the aim of the present study.

Our results show that the number of children and adolescents who required emergency psychiatric help for suicidal behaviors in the only 24-h emergency child and adolescent psychiatric service in Slovenia increased during the COVID-19 pandemic. The increase was shown only after a year of the pandemic and did not seem to directly correspond to the school closures. Longitudinal studies are needed to better understand the long-term consequences of the pandemic on adolescent suicidality and mental health.

## Data Availability Statement

The raw data supporting the conclusions of this article will be made available by the authors, without undue reservation.

## Ethics Statement

The studies involving human participants were reviewed and approved by Ethical Review Board of the University Psychiatric Clinic Ljubljana. Written informed consent from the participants' legal guardian/next of kin was not required to participate in this study in accordance with the national legislation and the institutional requirements.

## Author Contributions

MDR designed and conceived the study, analyzed, presented and interpreted the data, had full access to all the data in the study, and takes responsibility for the integrity of the data and the accuracy of the data analysis. BK, LLN, PL, and MDR were involved in the acquisition and interpretation of data. The first draft of the paper was written by BK and MDR. All the authors contributed to and approved the final version of the manuscript.

## Funding

The study was funded by the Slovenian Research Agency grant P3-0343.

## Conflict of Interest

The authors declare that the research was conducted in the absence of any commercial or financial relationships that could be construed as a potential conflict of interest.

## Publisher's Note

All claims expressed in this article are solely those of the authors and do not necessarily represent those of their affiliated organizations, or those of the publisher, the editors and the reviewers. Any product that may be evaluated in this article, or claim that may be made by its manufacturer, is not guaranteed or endorsed by the publisher.
